# Supplementary material for: All-at-once RNA folding with 3D motif prediction framed by evolutionary information
Source: Nat Methods. 2025 Oct 3;22(10):2094–106. doi: 10.1038/s41592-025-02833-w (PMC12510878; doi:10.1038/s41592-025-02833-w)
Supplement: Supplementary file 3 — Source code to run CaCoFold-R3D. Data and results. [file 41592_2025_2833_MOESM3_ESM.gz › supplemental_material/results/CaCoFold-R3D/CaCoFold-R3D_Rfam_vshuffle/RF02199_TTC28-AS1_2.R2R.sto.pdf]

RF02199_TTC28-AS1_2

5′- A A A A U R ● C A G A A ● G A R R G A A U ● A U R A R ● R R R A C U A G A G A R U A G G A A A G A C A ● ○ A A C Y R ● C R C Y C ● A A A ○ ○ A ● R A R R A A G G A C A U R U A A G A A A A A G A C ○ ○ ● U A C R Y R U G A A R A A A A ○ A G R C U R A U R G R U ● ● ● ● R U C Y C Y A U Y G U G U G A C A U Y U U C U U
